# Quality of public information matters in mate-choice copying in female zebra finches

**DOI:** 10.1186/s12983-015-0119-8

**Published:** 2015-10-01

**Authors:** Nina Kniel, Jennifer Schmitz, Klaudia Witte

**Affiliations:** Department of Chemistry and Biology, Institute of Biology, Research Group of Ecology and Behavioral Biology, Adolf-Reichwein-Str. 2, 57068 Siegen, Germany

**Keywords:** Artificial ornamentation, Mate-choice copying, Public information, Zebra finch

## Abstract

**Background:**

Mate-choice copying is a form of social learning in which an individual gains information about potential mates by observing conspecifics. However, it is still unknown what kind of information drives the decision of an individual to copy the mate choice of others. Among zebra finches (*Taeniopygia guttata castanotis*), only females (not males) copy the mate choice of others. We tested female zebra finches in a binary choice test where they, first, could choose between two males of different phenotypes: one unadorned male and one male artificially adorned with a red feather on the forehead. After this mate-choice test, females could observe a single unadorned male and a pair of zebra finches, i.e. a wild-type female and her adorned mate. Pair interactions were either restricted to acoustic and visual communication (clear glass screen between pair mates) or acoustic communication alone (opaque screen between pair mates). After the observation period, females could again choose between new males of the two phenotypes in a second mate-choice test.

**Results:**

In experiments with a clear glass screen, time spent with the respective males changed between the two mate-choice tests, and females preferred adorned over unadorned males during the second mate-choice test. In experiments with an opaque screen, time spent with the respective males did not change between the two mate-choice tests, although females lost an initial preference for unadorned males.

**Conclusions:**

Our results demonstrate that the quality of the received public information (visual and acoustic interaction of the observed pair) influences mate-choice copying in female zebra finches.

**Electronic supplementary material:**

The online version of this article (doi:10.1186/s12983-015-0119-8) contains supplementary material, which is available to authorized users.

## Background

Mate-choice copying is an important form of social learning in an intersexual context [1–3]. Females copy the mate choice or mate rejection of other females by observing a sexual interaction between a female and a male, and afterwards choose or reject that same male as the observed female did before [4, 5]. During mate-choice copying females gain supplemental information by observing other females and use this public information for their own mate-choice decisions [6, 7]. The influence of public information on female mate choice can be so strong that socially acquired information overwrites genetically based preferences for certain male phenotypes in females (e.g. [8–10]). Females do not only copy the choice for individual males, but they generalise and prefer other males of the same phenotype as mates as the observed preferred males as well [10–12]. Mate-choice copying has been experimentally demonstrated in several species, including birds (e.g. [12–19]).

Despite mate-choice copying being a widespread phenomenon, little is known about the mechanisms underlying this process. White and Galef [16] conducted experiments investigating this mechanism in the Japanese quail (*Coturnix coturnix japonica*) in which they previously found mate-choice copying by females [15]. Females showed mate-choice copying if a previously non-preferred male was courting a model female without being able to actually mate because of a partition keeping them apart (allowing visual and acoustic communication but no copulation). Females also copied if the observed male and the female were additionally separated visually from each other (no courtship/copulation, only proximity), but they did not change their choice if the observed male could court a model female but the observing female could not see the model female. Hence, the change in male behaviour was not the reason for the change in mate choice by female quail, but the proximity of a female to a male seemed to be sufficient for females to copy. However, to our knowledge, nothing is known about this mechanism in a socially monogamous system with biparental brood care and copying of a phenotype instead of an individual. For instance, it is not known what information is most important and what exactly females need to observe in order to copy. Is a full courtship display including a copulation necessary, or are more subtle cues like acoustic communication between a male and a female sufficient for copying the mate choice?

We used the zebra finch (*Taeniopygia guttata castanotis*) in the present study to investigate what quality of public information females need to copy the mate choice of female conspecifics. In female zebra finches, several studies have found mate-choice copying ([12, 18, 19], but see [20]). In a previous study, Kniel et al. [12] found that female zebra finches copy the mate choice of other females, but that males do not show mate-choice copying. Here, females generalised between males, i.e. they copied the choice for a specific male phenotype. In their study, they could also exclude other explanations for the change in mate choice in females. Based on these previous experiments, we manipulated the quality of the public information (observed pair could not interact physically, but could either communicate visually and acoustically, or only acoustically) that observing females received during mate choice in this study.

The zebra finch is a highly social species and lives in large flocks throughout the whole year. Pairs are inseparable in both breeding and non-breeding seasons [21]. However, since zebra finches are highly social and spend time together in colonies, proximity of a male and a female alone will most likely not be sufficient to identify a male and a female as a pair. Tactile interactions such as allopreening occur between pairs but also between other individuals of the flock [21]. Males may approach any female in a colony and court her, independent of whether she is already paired or not.

Obvious information about the choices of other females might only be available when the observing females witness a courtship interaction that ends in a copulation with female cooperation, or if they observe a pair during nest building and brood care. Potential partners cannot pair unless they can make tactile contact [22]. Visual and auditory contact and auditory contact alone are insufficient for a pair bond to form [22, 23]. After pair formation, pair bonds can be maintained when the partners are physically (or physically and visually) separated from each other but can still communicate acoustically [22]. Additionally, Immelmann [24] found that pairs can recognise their partner based on auditory cues alone. However, Galoch and Bischof [25] found in males that their choices for videos of unknown females over videos of their mates were based mainly on visual, not auditory cues. In contrast to that, females preferred their mates over unknown males, and their choices were affected by the manipulation of auditory cues [25], i.e. if auditory channels of the two presented male videos were switched, females lost their preference for their partner. This suggests that acoustic communication is important and may bear information for female observers.

We gave females the opportunity to observe a pair in two different situations. In one treatment, females could observe a pair of mated zebra finches that was able to communicate visually and acoustically, but not able to interact physically (pair was separated by a clear glass screen) during the observation period. In the other treatment, the pair could only communicate acoustically, and could neither communicate visually nor interact physically (pair was separated by an opaque screen). For our experiments, we used artificial ornamentation. Stimulus males were either adorned with a red feather, standing upright like a crest, or equipped with a piece of a grey flight feather (unadorned), representing the common phenotype. During the observation, we presented a pair, i.e. an adorned male and a wild-type female in one cage and a single unadorned male in another cage. We compared females’ choices for males of the two artificial phenotypes before and after the respective observation periods and tested whether they copied the mate-choice decisions of their conspecifics for the new adorned phenotype. Artificial ornamentation was used successfully to study a number of questions in the zebra finch [12, 18, 19, 26–31], and we used it in this study to be able to compare our results with previous findings on mate-choice copying in the zebra finch [12]. Depending on the importance of physical interaction of the observed pair for mate-choice copying, which was prohibited in both treatments, we would expect females not to copy in either case. On the other hand, acoustic communication is known to be sufficient for a pair to maintain their pair bond [22]. If this communication can be picked up by the observing females, and if this communication bears enough information, we might expect mate-choice copying to occur in both treatments. And if acoustic communication alone is not sufficient and additionally a visual interaction, without physical interaction, is needed, then we would expect them to copy in the treatment with a clear glass screen, but not in the treatment with an opaque screen.

## Results

Of the 34 females tested, four females showed a side bias during the first mate-choice test, after which the experiments were stopped. Of the rest, 15 females observed the pair separated by a clear glass screen and 15 females observed the pair separated by an opaque plastic screen. For male singing activity, results were missing for two experiments in each treatment. Therefore, comparisons were made with 13 experiments per treatment.

### Treatment one: experiments with a clear glass screen

**Fig. 1:**
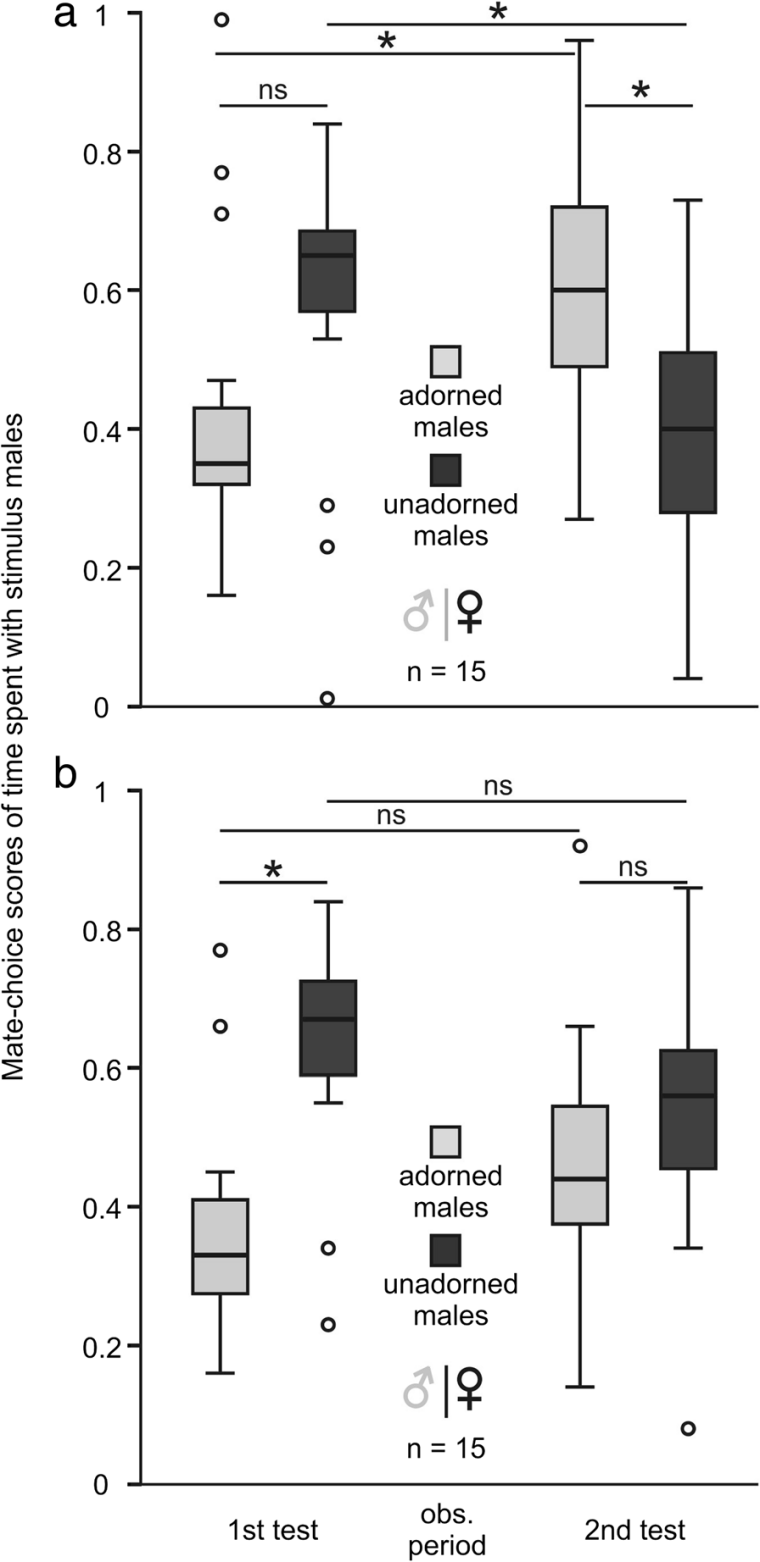
Results. Experiments with **a** a clear glass screen (grey line) and **b** an opaque screen (*black line*). Box plot showing median, first and third quartile, 95 % confidence limits and open points as outliers for mate-choice scores of time spent with adorned males (*grey bars*) and unadorned males (*black bars*). 1st test = first mate-choice test, 2nd test = second mate-choice test, obs. period = observation period, * = significant difference, ns = no significant difference

Choosing motivation (total time spent in both mate-choice zones during the 2 × 20 min mate-choice test) did not change between the first and the second mate-choice test (Wilcoxon-test: Z = −0.70, *N* = 15, *p* = 0.460). Mate-choice scores of time spent with adorned males were affected by test number (rmANOVA: F_1,14_ = 10.474, *p* = 0.006; Fig. 1a). Females spent more time with adorned males and less time with unadorned males during the second than during the first mate-choice test. Females showed no preference for one of the two males during the first mate-choice test (one-sample t-test: t = −0.800, df = 14, *p* = 0.437). However, excluding one extreme case in which a female spent only 20 s with the unadorned male while spending nearly 2000 s with the adorned male during the first mate-choice test, females showed a significant preference for unadorned males during the first mate-choice test (one-sample t-test: t = −2.352, df = 13, *p* = 0.035). Females preferred adorned over unadorned males during the second mate-choice test (one-sample t-test: t = −2.323, df = 14, *p* = 0.036). Pairs of stimulus males in all three steps of the experiment (*p* ≥ 0.312), as well as test females and stimulus females (unpaired t-test: t = 0.060, df = 28, *p* = 0.952), did not differ in weight (see Additional file 1). Adorned and unadorned males spent a similar amount of time in proximity to the test females in both mate-choice tests (*p* ≥ 0.461) (see Additional file 1) and sang a similar amount of times in both mate-choice tests (*p* ≥ 0.699) (see Additional file 1).

### Treatment two: experiments with an opaque plastic screen

Choosing motivation did not change between the first and the second mate-choice test (Wilcoxon-test: Z = −4.60, N = 15, *p* = 0.112). Mate-choice scores of time spent with adorned males were not affected by test number (rmANOVA: *F*_1,14_ = 1.9, *p* = 0.190; Fig. 1b). Females preferred unadorned over adorned males during the first mate-choice test (one-sample t-test: t = −3.094, df = 14, *p* = 0.008), but lost this preference during the second mate-choice test (one-sample t-test: t = −0.577, df = 14, *p* = 0.573). Pairs of stimulus males in all three steps of the experiment (*p* ≥ 0.227), as well as test females and stimulus females (unpaired t-test: t = 1.698, df = 28, *p* = 0.101), did not differ in weight (see Additional file 1). Adorned and unadorned males spent a similar amount of time in proximity to the test females in both mate-choice tests (*p* ≥ 0.089) (see Additional file 1) and sang a similar amount of times in both mate-choice tests (*p* ≥ 0.752) (see Additional file 1).

## Discussion

In our experiments, we tested whether female zebra finches would copy the choice for a certain male phenotype (artificial red feather on the forehead) if the quality of the received public information during an observation, i.e. the amount of interaction of a pair (adorned male with wild-type mate), was manipulated. We conducted two treatments in which the physical interaction of the pair was prohibited. In one treatment the interaction of the pair mates was restricted to acoustic and visual communication (clear glass screen between the observed pair mates), and in the other to acoustic communication alone (opaque screen between the observed pair mates). Females showed mate-choice copying if the observed pair could communicate visually and acoustically, but not if the pair could only communicate acoustically.

In experiments with the clear glass screen, females first showed no preference for one of the two male phenotypes, although an additional test without the female that spent nearly no time with the unadorned male at all, showed that the remaining 14 females had a preference for unadorned males. After having observed a pair that was able to interact acoustically and visually, but not physically, females spent more time with adorned males and less time with unadorned males during the second mate-choice test compared to the first mate-choice test. This change resulted in a preference for adorned males during the second mate-choice test. These results are consistent with the mate-choice copying experiments in Japanese quail by White and Galef [16] where the male was able to court the female but not to copulate with her, and also with those in zebra finches by Kniel et al. [12] where the observed pair could physically interact. We compared females’ mate choice after they had obtained public information (their mate-choice scores for adorned males during the second mate-choice test) with the results of the mate-choice copying experiment in Kniel et al. [12], where the pair mates could freely interact with each other, and our two treatments respectively. Females of our treatment with the clear glass screen showed a similar choice for adorned over unadorned males during the second mate-choice test (unpaired t-test: t = −0.744, df = 37, *p* = 0.462) as females in Kniel et al. [12]. In contrast to that, females of our treatment with the opaque screen showed a different choice during the second mate-choice test (unpaired t-test: t = −3.585, df = 37, *p* = 0.001) compared to females in Kniel et al. [12], i.e. they showed no preference for one of the two males. Our results showed that females have clearly been influenced in their mate choice by the information they could receive, which was a visual and acoustic interaction of a pair without physical interaction. In the experiment with a clear glass screen, both pair mates were able to actively seek the proximity of their mates by sitting on the edge of the perch at the glass screen. Males were able to sing directed at their mates and court them. Females could show tail-quivering, which is part of their courtship and the key sign for males that females are ready to mate with them [21]. However, physical interactions were restricted, i. e. the full courtship behaviour including a copulation, or social pair interactions like preening were not possible. The fact that females copied in this situation demonstrates that they do not need to observe the physical interaction.

In experiments with the opaque screen, where the observed pair was only able to interact acoustically, but neither visually nor physically, females showed no preference for either male during the second mate-choice test. They did not copy the mate choice for the adorned male phenotype. These results are not consistent with those found in female Japanese quail by White and Galef [16], who found that proximity (only acoustic communication allowed) of a previously non-preferred male to a model female subsequently made this male more attractive for the observing female. However, the Japanese quail and the zebra finch differ in their mating system. While the zebra finch is socially monogamous, female Japanese quail are promiscuous and may mate with more than one male [32]. First of all, this difference is reflected in the fact that female zebra finches only copy the choice for a male phenotype and not for an individual ([12, 18, 19], but see [20]), while female quails readily copy the choice for individual males [15–17]. Second, because of these differences, social information about pair bond behaviour rather than proximity alone is likely to be more important in zebra finches than in Japanese quail. Butterfield [33] provided evidence that visual stimuli are important in bond maintenance in zebra finches. However, Silcox and Evans [22] found that a pair bond in zebra finches could be maintained if the pair was only allowed to have acoustic contact. And Miller [34] stated that zebra finch mates learn to recognise one another’s vocalisations. It seems that observing females were not able to recognise these cues as the communication of a pair.

One explanation for the absence of mate-choice copying in experiments with the opaque screen could be inconsistency in mate choice. However, previous experiments have already demonstrated that females choose consistently when they have no opportunity to copy [12]. Additionally, in one of the controls performed by Kniel et al. [12], where females lost an initial preference for unadorned males, there was no pair present during the observation period, only a single male. Hence, females could not have picked up public information, but still lost their preference. Therefore, acoustic cues might bear some information, but it seems as if this information is not sufficient for females to copy mate choice. Additionally, in contrast to the study in Japanese quail [16], the mere proximity of a male and a female does not seem to be sufficient for observing females to copy the mate choice of their conspecifics, there must be some amount of interaction. This makes sense, since the zebra finch is a highly social species that lives in large flocks throughout the whole year. There will always be males and females close to each other that are not a pair and, therefore, proximity of two birds alone will not be sufficient to recognise them as a pair. And since the zebra finch is quite communicative [21], it might be difficult to extract information from acoustic communication alone.

Since the weight of stimulus males, time spent close to the test females, and song did not differ between the respective stimulus males, we can assume that stimulus males differed only in their artificial ornamentation. Additionally, stimulus females had a similar weight as test females. Females in our experiments are, therefore, believed to have based their preference during the first mate-choice test on the artificial ornamentation of the stimulus males, and their change of preference on the quality of the available public information during the respective observation periods.

Our experiments demonstrate that mate-choice copying in the zebra finch depends on the quality of the public information received by observing females. First, we found that the information that females extract from a visual and acoustic interaction of a pair bears the information needed for them to copy the mate choice of their conspecifics. Second, results demonstrate that proximity and/or acoustic communication of the observed pair does not seem to be sufficient for females to copy. And third, we could show that physical interaction of the observed pair is not needed. This is a first insight into a part of the mechanism of mate-choice copying and what quality of public information matters in mate-choice copying for female zebra finches.

## Conclusions

Although mate-choice copying has been investigated in different species and in a number of contexts, it is still widely unknown what kind of information, i.e. quality of public information, drives the decision of an individual to copy the mate choice of others. We found that female zebra finches showed mate-choice copying if the received public information included visual and acoustic communication of the observed pair, but no physical interaction, e.g. social interactions like allopreening or a copulation. Additionally, we found that acoustic communication alone was not sufficient. This demonstrates that the quality of the received public information influences mate-choice copying in female zebra finches, i.e. whether or not females copy, and gives a first insight into what quality of public information actually matters for mate-choice copying.

## Methods

### Study species

Test and stimulus birds were sexually mature F_7–9_ descendants (females: mean age about 31 months, minimum: 8 months, maximum: 46 months; males: mean age about 30 months, minimum: 8 months, maximum: 44 months) of wild zebra finches that were exported from Northern Victoria, Australia, in 1992 (Meyer T, personal communication). They were kept in five aviaries (four: 2 × 1.65 × 2.30 m^3^, one: 2.25 × 1.05 × 2.30 m^3^), separated by sex after maturation (mean 71, minimum 56, and maximum 92 days after hatching) for at least six months before the experiments. The air-conditioned room (6.80 × 4 × 2.40 m^3^) (Temperature = 24° ± 1 °C, Humidity = 60 % ± 10 %) with windows at two sides was illuminated with fluorescent lighting including UV-range at a 14:10 h light:dark photoperiod. Both sexes wore numbered orange or white leg bands, or silver metal leg bands (neutral in zebra finch mate choice; [27, 28, 35]. Each aviary contained several branches, coconut fibres for nest building, several nest-boxes, and sand, food and water *ad libitum*. Zebra finches were fed daily with a mixture of seeds containing Senegal, red, yellow and Canary millets; sprouted birdseed; and cucumber, chickweed, and crunched eggshells.

All behavioral experiments were performed under the permission of the County Veterinary Office, Siegen, Germany (permit numbers: 53.6 55-05). We declare that this study was carried out in strict accordance with the recommendations in the Guide for the Care and Use of Laboratory Animals of the German Right of Animal Welfare (Tierschutzgesetz).

### Experimental design

**Fig. 2:**
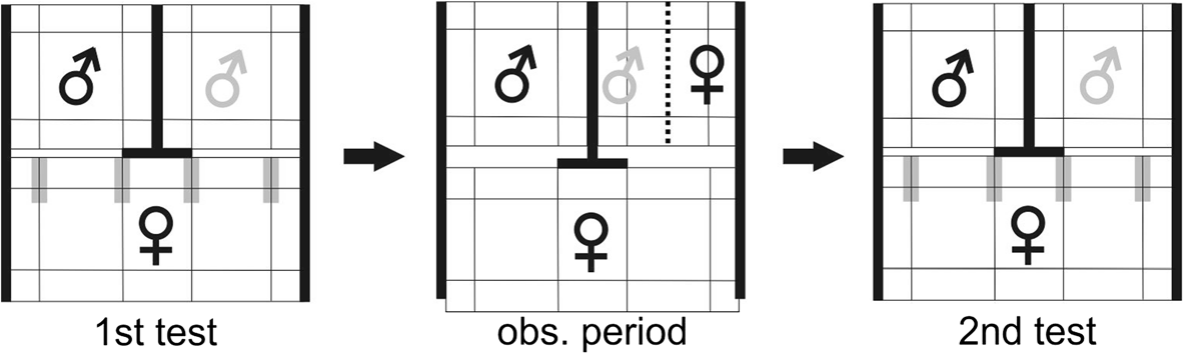
Experimental setup. Experimental setup, top-view. Grey zones are mate-choice zones. Grey male symbol = adorned male, black male symbol = unadorned male. Bold bars are opaque screens. Thin lines within the cages represent perches. The dotted line represents the clear glass screen (the opaque screen was positioned in the same way). 1st test = first mate-choice test, obs. period = observation period, 2nd test = second mate-choice test

Experiments were conducted in November and December 2010 in an air-conditioned room without windows (2.20 × 2.10 × 2.40 m^3^) under the same conditions as in the aviary room. The mate-choice copying experiments were performed in cages (49 × 43 × 50 cm^3^); stimulus males were placed side by side, and the cage of the test female (97 × 43 × 52 cm^3^) was placed in front of them (Fig. 2). Each cage contained water, food, and sand *ad libitum* in little bowls on the ground, and four perches: one low perch parallel and near to the front (10 cm above the bottom of the cage), one high perch parallel and near to the backside (35 cm) and two additional perches parallel to the side of the cage in middle height (20 cm). The cage of the test female had two additional perches of choice in middle height. The cage of the pair was constructed slightly different. In the middle of the cage, on the ground and under the ceiling, we installed a plastic guide rail. The wire mesh on the back of the cage was separated in half and allowed enough space to enter a screen, guided by the plastic rail. In treatment one, we used a clear glass screen, in treatment two we used a grey opaque plastic screen. These screens divided the cage in two halves. Perches were fixed at the same height and position as in the other cages, but the two perches parallel to the front of the cage were divided in two halves. They were fixed at the ceiling with square-shaped timber (diameter 1.5 cm). This allowed us to keep the pair in the same cage, but separate them with a screen. Both screens prevented physical interactions, although the male and the female could sit close to each other. The clear screen allowed visual and acoustic communication, whereas the opaque screen additionally prevented visual communication and only allowed acoustic communication.

A wooden screen, placed between the stimulus cages, prevented visual contact between the stimulus males. An additional screen (18 cm wide and 49 cm high), located at the front and in the middle of the test female’s cage, prevented the test female from seeing both stimulus males when being in direct proximity of one of the male cages.

All birds were kept in test or stimulus cages at least 15 h before we started the experiments the next morning in visual but not acoustic isolation from other birds. Stimulus males were either adorned with a red feather, standing upright like a crest and representing a conspicuous trait, or equipped with a piece of a grey flight feather (unadorned), representing the common phenotype, when they were caught. Red feathers were cut out of a red feather boa along the quill (length: 2 cm, width: 4–5 mm). Grey flight feathers were cut to triangles (maximum edge length 5 mm). Both were glued to the forehead with double-sided tape onto their natural forehead feathers, which could easily be removed afterwards. This way, all stimulus males were handled equally and were not harmed. This method was used successfully in a number of experiments with zebra finches [12, 30, 31] and the Javanese mannikin *Lonchura leucogastroides* [36, 37]. 

### Procedure

In the first mate-choice test, test females could choose between an adorned (red feather) and an unadorned male to determine the initial mate preference and to test whether test females had a latent, that is, genetically determined, preference for the novel male phenotype. During the observation period, test females could observe new stimulus males, one single unadorned in one cage and one adorned male with his wild-type female mate in the other cage. The respective pairs were taken from their breeding cages and transferred to the stimulus cage. They had been together for several months and had reproduced with each other. After this observation period, test females again got the opportunity to choose between two new stimulus males, one adorned and one unadorned (second mate-choice test). Between the different phases, we gave birds the time to acclimate for at least 5 min.

By removing the screens, we started the first mate-choice test, which lasted 2 × 20 min with a switch of stimulus males’ cages after 20 min to control for side biases. We measured the time (s) the test females spent perching on the outer one-third of the perches of choice adjacent to the stimulus males (mate-choice zone; grey area in Fig. 2) every 10 s. If the test females changed position during the 10-s interval, 5 s were scored, otherwise 10 s. All other positions, which included the greater part of the cage (e.g. feeding on the ground or sitting on the other perches), were scored as no choice positions. Thus, the choice positions covered only 16 % of all possible perching positions. This method is an established measurement to determine sexual preferences in zebra finches [12, 38]. We calculated their choosing motivation (total time spent in both mate-choice zones during the 2 × 20 min mate-choice test). Additionally, we measured the time that the respective stimulus males spent in front of their cages (outer one-third of the perches close to the females), and the number of times that males sang. Male song rate is known to influence female mate choice as they spend more time with males that sing more often compared with those that sing less often [39]. During the observation period, which lasted 2 h, females could observe a new, single unadorned male in one cage and a pair of zebra finches, a new adorned male and his wild-type female mate separated by a screen, in the other cage. The side where the pair was presented was randomised. The second mate-choice test was performed like the first, but with new stimulus males. After each experiment we measured the body weight of all birds and placed them back into their aviaries or cages. We used each test female only once. Since the number of available birds was limited, we used stimulus males for three to four consecutive mate-choice tests, but always in different combinations and both as an adorned or an unadorned stimulus. Stimulus pairs were also reused. Test females were not closely related to stimulus birds, and stimulus males were not known to test females. In eight experiments of each treatment, the stimulus females were known to test females (same aviary), and in the other seven they were unknown to test females (different aviaries).

Throughout the whole testing time (10 min before starting the first mate-choice test until the last mate-choice test was over) we played zebra finch sounds (recorded in the aviary room in 2008) through a loudspeaker (Speed Link, Brave 2.0 Stereo Sound System). Since zebra finches live in flocks, they tend to be relatively inactive if they do not hear calls of conspecifics. We placed the loudspeakers on the ground, about 30 cm away from the table on which we placed the test female. The sound was played at about 60–70 dB, measurements depending on the type of sounds the birds made. This equals the sound pressure level in the middle of our aviary room.

Test females that showed a side bias during the first mate-choice test, those that spent more than 80 % of their choosing time on the same side, even though we switched the position of the stimulus cages, were excluded from the analysis in accordance with other studies [12, 40–43]. We tested a total number of 34 females.

### Statistical analysis

We analysed the time test females spent within the mate-choice zones in front of stimulus males. To analyse female choosing motivation, we used a Wilcoxon-test. We used mate-choice scores of time spent with the adorned stimulus (time spent with the adorned stimulus/time spent with both the adorned and the unadorned stimuli) and tested whether this was influenced by test number. We transformed mate-choice scores via arcsine-square-root to have normally distributed data and used a repeated-measures Anova (rmAnova) (with mate-choice test as within-subject factor). Where Anova results did not conform to the assumption of sphericity, Greenhouse-Geisser approximations were used. To test whether test females showed a preference for one of the two stimulus males, we tested the mate-choice scores of time spent with adorned males against a 50 % expectation using a one-sample t-test. To compare weight of birds we used an unpaired t-test. To compare the time that males spent close to test females, and the number of intervals in which males sang, we used a Mann-Whitney-U test. Statistical analyses were performed using SPSS (IBM SPSS Statistics 22). All *p* values are two-tailed.

## Additional file

Additional file 1:
**Table of weights, male time spent and male song.** The table contains the weight of birds used in the experiments, the time stimulus males spent close to the test females, and the number of times stimulus males sang within the two mate-choice tests. Given are the median, 1^st^ quartile and 3^rd^ quartile. adorned = adorned stimulus males, unadorned = unadorned stimulus males. (XLS 37 kb)
